# *Web-CONEXS*: an inroad to theoretical X-ray absorption spectroscopy

**DOI:** 10.1107/S1600577524005630

**Published:** 2024-08-01

**Authors:** Joshua D. Elliott, Victor Rogalev, Nigel Wilson, Mihai Duta, Christopher J. Reynolds, Jacob Filik, Thomas J. Penfold, Sofia Diaz-Moreno

**Affiliations:** a Diamond Light Source Harwell Science and Innovation Park Didcot OxfordshireOX11 8UQ United Kingdom; b Chemistry – School of Natural and Environmental Science, Newcastle University, Newcastle Upon-TyneNE1 7RU, United Kingdom; ESRF – The European Synchrotron, France

**Keywords:** *Web-CONEXS*, X-ray absorption spectroscopy, XAS, first-principles simulation, density functional theory, DFT, X-ray absorption near-edge structure, XANES

## Abstract

Accurate analysis of the rich information contained within X-ray spectra usually calls for detailed electronic structure theory simulations. However, density functional theory (DFT), time-dependent DFT and many-body perturbation theory calculations increasingly require the use of advanced codes running on high-performance computing (HPC) facilities. Consequently, many researchers who would like to augment their experimental work with such simulations are hampered by the compounding of nontrivial knowledge requirements, specialist training and significant time investment. To this end, we present *Web-CONEXS*, an intuitive graphical web application for democratizing electronic structure theory simulations. *Web-CONEXS* generates and submits simulation workflows for theoretical X-ray absorption and X-ray emission spectroscopy to a remote computing cluster. In the present form, *Web-CONEXS* interfaces with three software packages: *ORCA*, *FDMNES* and *Quantum ESPRESSO*, and an extensive materials database courtesy of the Materials Project API. These software packages have been selected to model diverse materials and properties. *Web-CONEXS* has been conceived with the novice user in mind; job submission is limited to a subset of simulation parameters. This ensures that much of the simulation complexity is lifted and preliminary theoretical results are generated faster. *Web-CONEXS* can be leveraged to support beam time proposals and serve as a platform for preliminary analysis of experimental data.

## Abstract 2

*Web-CONEXS* is introduced – a web application hosted on *ISPyB* at Diamond Light Source, which provides a platform for modelling XAS spectra based on minimal user input.

## 1. Introduction

X-ray spectroscopic capabilities at third- and fourth-generation light sources such as synchrotrons and X-ray free-electron lasers (XFELs) are increasing at a rapid rate due to the development and deployment of high-brilliance light sources and flexible modular beamlines, which permit highly detailed, complex, time-resolved investigations *in situ* and under *operando* conditions (Dent *et al.*, 2009 ▸; Diaz-Moreno *et al.*, 2009 ▸; Mosselmans *et al.*, 2009 ▸; Taylor *et al.*, 2013 ▸; Figueroa *et al.*, 2018 ▸; Diaz-Moreno *et al.*, 2018 ▸; Hayama *et al.*, 2021 ▸). X-ray spectroscopic data obtained on these beamlines can unravel both the structural and the electronic properties of technologically relevant systems across the chemical (Bartlett *et al.*, 2019 ▸; Boada *et al.*, 2022 ▸; Ma *et al.*, 2022 ▸), physical (Arias-Egido *et al.*, 2021 ▸; Nicholls *et al.*, 2022 ▸; Das *et al.*, 2022 ▸) and materials sciences (García *et al.*, 2021 ▸; Alkhalifah *et al.*, 2022 ▸; Foster *et al.*, 2022 ▸). These massive advances in technological capability increase the focus on the far-from-trivial task of analysing and interpreting the wealth of information into the electronic, geometric and spin structure of matter that X-ray spectra contain.

This challenge can be offset through theoretical materials modelling and simulations based on electronic structure theory (Timrov *et al.*, 2020 ▸; Kellett *et al.*, 2020 ▸; Groot *et al.*, 2021 ▸; Begum *et al.*, 2021 ▸; Rankine & Penfold, 2021 ▸). Theoretical support for X-ray spectroscopy comes in a range of different flavours which differ primarily in the balance between computational intensity and accuracy they offer: methods include atomic multiplet theory (Haverkort *et al.*, 2012 ▸), multiple scattering theory (MST), single-particle approaches based on *ab initio* electronic structure such as density functional theory (DFT) (Besley, 2021 ▸) and Hartree–Fock (HF) (Norman & Dreuw, 2018 ▸), and methods that investigate many-body phenomena such as time-dependent DFT [TD-DFT (Norman & Dreuw, 2018 ▸; Besley, 2021 ▸; Bussy & Hutter, 2021 ▸)], many-body perturbation theory [MBPT (Vinson *et al.*, 2011 ▸; Gilmore *et al.*, 2015 ▸; Vorwerk *et al.*, 2017 ▸, 2019 ▸)] as well as those based on correlated wavefunctions (Maganas *et al.*, 2017 ▸, 2018 ▸; Norman & Dreuw, 2018 ▸; Chantzis *et al.*, 2018 ▸; Pinjari *et al.*, 2016 ▸).

The development and curation of such a wide range of available theoretical approaches have ensured that theoretical X-ray spectroscopy has kept pace with experimental advances. However, for non-experts using X-ray spectroscopy as a characterization tool, this diversity in theoretical approaches can make it difficult to know where to start. To illustrate this point, consider a scenario in which X-ray absorption near-edge structure (XANES) spectra have been collected at a synchrotron facility, and electronic structure theory simulations could be employed to help interpret the data. To perform first-principles simulations, users must make several informed decisions which could serve as severe obstacles to those without experience or access to an experienced collaborator. In particular, for any given material, the user must select an appropriate method for the simulation. The method is one of the different flavours of theory outlined above. It is possible to draw on the literature to help with this selection, yet without an intuitive feel for (i) computational viability, (ii) the validity of underpinning physical approximations and (iii) subtle differences between similar systems that can lead to vastly different requirements, then it can be challenging to evaluate the transferability of previous work.

The selection of the method is only the first barrier; other constraints play a role in the limited uptake of theoretical modelling. The next choice is the specific calculator for the simulation. The calculator is the software or computer code that implements the physical equations and materials relationships. Again, usually, some direction can be taken from the literature; however, as in the case of point (iii), the user must also recognize that the nature of the material will also dictate the choice of the calculator as well as the availability of features across different software packages. For example, crystalline materials with well defined repeat unit cells lend themselves to calculators that implement periodic boundary conditions and basis sets based on plane waves such as *Quantum ESPRESSO* (*QE*), (Giannozzi *et al.*, 2009 ▸, 2017 ▸, 2020 ▸) *CASTEP* (Clark *et al.*, 2005 ▸) and *Exciting* (Gulans *et al.*, 2014 ▸). On the other hand, finite systems such as molecules and clusters of atoms can instead leverage a calculator that is based on localized (Gaussian) basis functions and open boundary conditions such as *ORCA* (Neese, 2012 ▸, 2018 ▸, 2022 ▸), *NWChem* (Valiev *et al.*, 2010 ▸; Apra *et al.*, 2020 ▸) and *QChem* (Epifanovsky *et al.*, 2021 ▸). In addition, the choice of calculator requires the user to fully understand the physical equations, including key approximations, which are implemented. This forces the user to develop a working knowledge of the mapping between the model and materials relationship and the set of code-specific input parameters. Usually, each calculator has its own set of input parameters and syntax, meaning the user must invest time to learn the unique calculator language. Finally, the user will likely need access to an advanced or high-performance computing (HPC) facility to conduct theoretical XAS simulations. However, note that this is only sometimes the case; software such as *FEFF* (Rehr *et al.*, 2010 ▸), *MXAN* (Benfatto *et al.*, 2021 ▸) and *FDMNES* (Bunău & Joly, 2009 ▸) (in Green’s function mode) that employ MST, and *Quanty*/*Crispy* which leverage semi-empirical atomic multiplet theory (Haverkort *et al.*, 2012 ▸), all circumvent HPC requirements. Yet software implementing higher-precision approaches like full potential DFT and beyond-DFT simulations demands HPC to go beyond crude small-scale models. Although gaining access to such a facility is another obstacle, the user must also understand and conform to the HPC syntax for running simulations within a batch job environment. This is, in fact, a larger issue than it appears at first glance because non-conformity may lead to improper and disruptive use of computing resources.

To summarize, a non-expert user faces four critical obstacles before running *ab initio* simulations: choice of model, choice of calculator, familiarity with calculator language and access and familiarity with HPC. To address these obstacles, in this work, we introduce *Web-CONEXS*, an intuitive and user-friendly web application deployed on the Diamond Light Source (DLS) * ISPyB* platform. *Web-CONEXS* mitigates many difficulties associated with *ab initio* simulations by directly interfacing with calculators and the HPC facility. It tackles challenges with the choice of model and calculator by limiting the number of available options, thereby reducing the number of decisions the user has to make. The integrated calculators have been tailored so that they can be selected according to molecular or crystalline materials, with a third option for large systems. *Web-CONEXS* provides a viable minimal set of input parameters for any given material and calculator, which can be used as a simulation starting point. Finally, *Web-CONEXS* automates job submissions and returns the results to the user, ultimately providing the foundation for further computation.

This manuscript is organized as follows: in the next section, we outline the *Web-CONEXS* workflow from the user perspective and based on the machinery hidden from the user. We then provide technical details on the implementation and discuss each interfaced calculator. In the final section, we highlight a case study for each calculator and describe how to build on the *Web-CONEXS* example to achieve production-level simulation results.

## 2. Implementation

*Web-CONEXS* has been conceived and implemented with the non-specialist user in mind. Its target is to provide support for users with limited or no experience of simulation and HPC to gain experience which can subsequently be transferred to independent use of HPC. It is not expected to be used by researchers with experience in the simulation of materials using electronic structure-based approaches or for detailed analysis of data. *Web-CONEXS* can be accessed by past and present DLS synchrotron users through the *ISPyB* experiment portal.

### 2.1. *Web-CONEXS* workflow

**Figure 1:**
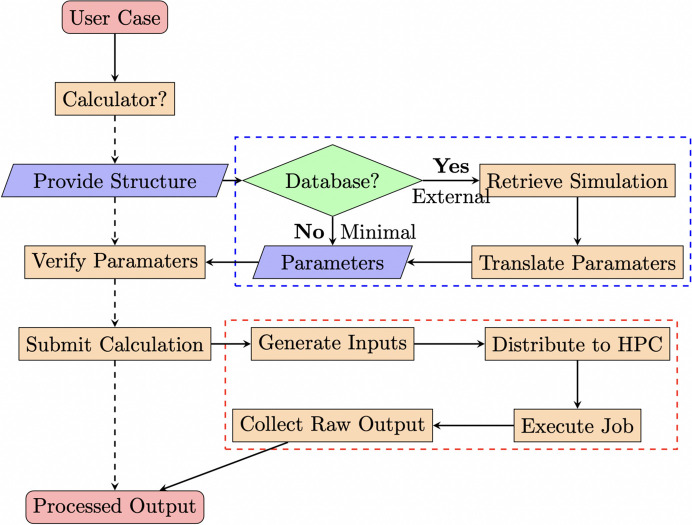
Schematic of the *Web-CONEXS* user simulation workflow. Dashed arrows denote the steps taken by a *Web-CONEXS* user, solid arrows denote steps hidden from the user. Blue and red dashed boxes group together steps hidden from the user: in blue are the steps taken to build the input file and in red are the steps taken to execute the calculation.

This section outlines the workflow for an XAS simulation carried out on the *Web-CONEXS* platform. Fig. 1 ▸ depicts a flowchart for the process. Steps that relate directly to the user are connected with dashed arrows and steps hidden from the user are connected with solid arrows.

The first step for the user is selecting one of the available calculators. These calculators are external software packages that implement the physical equations and materials relationships. Currently, *Web-CONEXS* supports the following calculators: *ORCA* (Neese, 2012 ▸, 2018 ▸, 2022 ▸) for DFT and TD-DFT on molecular systems and cluster type calculations, *FDMNES* (Joly, 2001 ▸; Joly *et al.*, 2009 ▸; Bunău *et al.*, 2021 ▸) for finite differences and Green’s functions approaches, and *QE* (Giannozzi *et al.*, 2009 ▸, 2017 ▸, 2020 ▸) for DFT on periodic systems. Further details on the supported features for each calculator are provided in the following section.

On the *Web-CONEXS* platform, a user case can be fully described using atomic coordinates and unit-cell vectors for crystalline systems. The user is asked to provide the atomic coordinates atom-by-atom, by reading an existing file in either .xyz or .cif format, or through a calculator-specific input file. In some instances, atomic coordinates can be provided by a reference to an external database of simulation data: *Web-CONEXS* currently interfaces to the Materials Project API (Jain *et al.*, 2013 ▸; Ong *et al.*, 2015 ▸), which allows for the definition of a vast catalogue of experimental and theoretical materials structures based solely on a materials project identification code. Based on the input structure, *Web-CONEXS* returns a sensible, minimal set of simulation parameters for the user to verify. The permitted input options for each calculator have been limited to avoid overly complex and excessively long calculations, which are outside the remit of *Web-CONEXS*. A detailed list of the allowed parameters is provided in the supporting information. In addition to the calculator parameters, there are also compute options related to the batch job scheduler. These options control the calculations’ size and the workload distribution across compute cores working in parallel. *Web-CONEXS* sets these options automatically to decrease the demand on the user knowledge requirement of specific HPC architecture and syntax.

The user can then submit the calculation to a computing facility for execution. During the simulation, the user can query the batch job status and stop the simulation at any point through the *Web-CONEXS* user interface. On completion, *Web-CONEXS* processes the raw output and returns a text file containing energy versus intensity via email to the user.

In the background, the user workflow is augmented by several steps, denoted by solid arrows and grouped by the blue and red dashed boxes in Fig. 1 ▸. Firstly, on inputting the atomic coordinates, *Web-CONEXS* takes several steps to provide the set of minimal input parameters. For *QE* calculations, the user may want to provide a reference to their material from the Materials Project database (Jain *et al.*, 2013 ▸; Ong *et al.*, 2015 ▸). In this case, not only can the coordinates be derived from the database reference, but also a guess of the calculator (*QE*) input parameters, which can be derived from the first-principles simulations also stored in the database. These externally sourced parameters can be translated to the *QE* namespace before being presented to the user. In practice, this leads to overly complex input files and simulations that exceed the compute resources currently available to *Web-CONEXS*. In the present implementation, *Web-CONEXS* derives the system geometry, lattice vectors and the *k*-point mesh used to sample the first Brillouin zone from the Materials Project database entries. Conversely, *Web-CONEXS* uses system information to determine the minimal calculator parameters if the user supplies coordinates.

When the user submits a compute job, the job is scheduled to run on resources that are part of the IRIS infrastructure. IRIS is a collaborative project that aims to provide computing infrastructure to DLS by allocating CPU cores, storage and GPUs. The *Web-CONEXS* project has a resource allocation of 690 CPUs and 2.9 TB of memory. The computing infrastructure is hosted by the STFC, which provides resources using an Openstack based Cloud. These consist of virtual machines (VMs), object storage and block storage. The IRIS infrastructure is virtualized and suitable for high-throughput computing (HTC), but it does not provide high-performance/low-latency storage or networks and, hence, is not an HPC. To facilitate these workloads inside DLS, a workload scheduler different from the *Gridengine* based system is used. This system is called *HTCondor*.

*HTCondor* has semantics similar to *Gridengine*, plus a few extra features that make it ideal in a Cloud environment. For example, it has the notion of jobs (which can be started and stopped), input files that *HTCondor* can transfer to the location of the running job and output files which *HTCondor* can return to the user. *HTCondor* submits jobs to a submitter via ssh or Python API. The submitter matches the job to resources advertised by the worker nodes or ‘executors’ in *HTCondor* terminology. The *HTCondor* workers run on IRIS VMs, whilst the submitter runs inside DLS.

Before the job is finalized and submitted to the *HTCondor* submitter, the *Web-CONEXS* backend generates any files needed for the calculator’s execution and for reporting the results. These include input files for a chosen calculator, instructions to run a containerized calculator(s), and transfer input and output files. Containerization carries the benefit that each simulation carried out on the *Web-CONEXS* platform is packaged, fully portable and reproducible across any computing architecture at any point in time. On completion, output files from the calculator are transferred back by *HTCondor* and then returned to the user as an email.

### 2.2. Calculators

#### 2.2.1. ORCA

*ORCA* is a quantum chemistry software package that performs simulations in open boundary conditions (Neese, 2012 ▸, 2018 ▸, 2022 ▸). This makes it an ideal calculator for molecules, clusters and nanoparticles. By default, *ORCA* employs Gaussian basis sets for expanding the electronic wavefunctions and can treat materials using an all-electron approach or with pseudopotentials. To calculate the XAS spectrum at the *K*- and *L*-edges, *ORCA* implements both TD-DFT and correlated wavefunction-based methods, including configuration interaction singles (CIS), complete active space self-consistent field (CASSCF) and coupled cluster theory, including singles and doubles (CCSD) (Maganas *et al.*, 2017 ▸, 2018 ▸; Chantzis *et al.*, 2018 ▸).

On the *Web-CONEXS* platform, it is possible to compute the XAS and XES spectra with *ORCA* using TD-DFT and DFT, respectively; the workflow for such a calculation is straightforward. In one step, the spectrum can be calculated by specifying a TD-DFT or DFT simulation and fixing the excitation range from one of the core orbitals to the virtual orbitals. To do this the user is only required to provide structural information and then select from appropriate options on the interface for the system they will investigate. This structure may be loaded from an .xyz or .gmnt file, or inserted manually as Cartesian coordinates. For an *ORCA* simulation, the user should specify the total charge and spin multiplicity of the system, and the orbitals expected to participate in the X-ray transitions. In other words, the orbital range that describes the initial and final states for absorption.

Additional and more specialist options that the user is requested to provide are the basis set, exchange-correlation potential and solvent environment. To aid in this selection, *Web-CONEXS* limits the choice to three basis sets and three exchange-correlation potentials. These have been chosen to provide a reasonable first guess at the XAS/XES spectrum in most cases, in particular with the option to select the triple zeta def2-TZVP basis set for the simulation of higher-*Z* elements in the periodic table. Further details can be found in the supporting information. In XES mode, the acceptor core orbitals are set to the lowest-energy orbitals, spin channels to α and β and with spin-orbit coupling interactions are turned on. Note that in some cases the transitions of interest could be to higher energy orbitals. In this instance, the user must manually modify the core orbital within the input and perform an additional calculation outside the platform.

#### 2.2.2. Quantum ESPRESSO

*Web-CONEXS* provides an interface to the pw.x and xspectra.x codes of the *QE* software suite for the simulation of ordered crystalline systems (Giannozzi *et al.*, 2009 ▸, 2017 ▸, 2020 ▸). *QE* is a collaborative open-source project implementing Kohn–Sham DFT, expanding electronic wavefunctions using a plane wave basis set and treating the highly oscillatory core level states within the pseudopotential approximation. The *XSpectra* package (Taillefumier *et al.*, 2002 ▸; Gougoussis *et al.*, 2009*a* ▸,*b* ▸; Bunău & Calandra, 2013 ▸), which is utilized to simulate core-level excitations, computes the XAS spectrum at the *K*- and *L*-edges by solving Fermi’s Golden rule with the recursive Lanczos–Haydock extended fraction approach. For the core levels that act as the initial states for the excitation, *XSpectra* leverages Blochl’s projector augmented wave (PAW) method to recover all-electron wavefunctions (Blöchl, 1994 ▸).

Obtaining the XAS spectrum with *QE* is a multistep process; in the simplest case, the first step is to perform a single-point self-consistent field calculation to compute the electronic density of the ground state. This is done with the pw.x code. The second step is to use the Lanczos–Haydock algorithm to calculate the absorption spectrum, within the dipole approximation, for a given atom in the system using the xspectra.x code. The *QE* approach also offers additional complexity by including a (fractional) core-hole potential (Bunău & Calandra, 2013 ▸), anisotropic polarization of the transition matrix operator (Taillefumier *et al.*, 2002 ▸), the calculation of quadrupole contributions to the spectrum (Gougoussis *et al.*, 2009 ▸*b*) and several different Hubbard correction schemes (Gougoussis *et al.*, 2009 ▸*b*; Timrov *et al.*, 2020 ▸). Though *Web-CONEXS* can undoubtedly be an entry point for these more complex simulations, they remain outside the scope of the platform.

The minimum requirements for calculating the XAS spectrum within the *QE* software suite are a description of the structure, a kinetic energy cutoff that defines the size of the plane wave basis set and a set of Gauge-including PAW (GIPAW) pseudopotentials (Pickard & Mauri, 2001 ▸) for describing the core levels. The user must provide the crystal structure for their material in Cartesian coordinates; this may be provided on an atom-by-atom basis or by reading in a .xyz file. The user must also provide the set of crystal lattice vectors. The *Web-CONEXSQE* interface also provides access to the Materials Project database through the Materials API (MAPI); structural information may be imported directly to *Web-CONEXS* by providing a Materials Project ID number. The size of the basis set and the description of the core electrons are fixed in *Web-CONEXS*, which includes the complete set of PBESol pseudopotentials from A. Dal Corso’s PSlibrary (version 1.0.0; Dal Corso, 2014 ▸) modified to include GIPAW reconstructions for each element. Furthermore, the user can select from a set of predefined input parameters depending on whether the material of interest is a metal, semiconductor or insulator; more details relating to these parameters are provided in the supporting information. For the XAS calculations, the user can then select the absorbing species and edge to be computed based on the input structure.

#### 2.2.3. FDMNES

For the simulation of large molecular, crystalline or low-dimensional systems, *Web-CONEXS* has an interface to the *FDMNES* code (Joly, 2001 ▸; Joly *et al.*, 2009 ▸; Bunău *et al.*, 2021 ▸). *FDMNES* can be described as user-friendly software for the computation of the XANES spectrum through the solution of Fermi’s Golden rule up to and including electronic octupole and magnetic dipole transitions, implementing the fully relativistic local spin density approximation (Joly, 2001 ▸). *FDMNES* performs these calculations in real space, building clusters (of user-defined size) around absorbing atoms in the molecule or unit cell. Of particular importance, *FDMNES* offers two methods for the computation of the spectrum: first, with MST, which provides a way to rapidly compute (at low precision) the spectrum with Green’s functions; and second, with a finite difference method (FDM) approach. Though computationally efficient and not requiring access to HPC, MST calculations rely on the Green’s function formalism on top of a muffin-tin (MT) potential that can result in limited agreement with experimental spectra. Instead, the FDM overcomes limitations associated with the MT approximation, allowing for an unconstrained potential to lead to better agreement with experimental measurements at the cost of computational complexity and the requirement for HPC.

A typical simulation requires as input: the structure of the system to be investigated, the type of calculation to be performed (either MST or FDM), the size of the cluster around the absorbing atoms, and the absorbing element and edge that will be computed. Many other input parameters may be added or modified to systematically improve the simulation spectrum with respect to the experimental one (Joly *et al.*, 2009 ▸). Within *Web-CONEXS*, the user must provide information on the structure of their system. Geometric data may be provided from an existing file; currently supported file formats are .cif and .pdb. Alternatively, the atomic coordinates (Cartesian) and simulation cell data may be entered manually. Additionally, the user must specify either the MST or the FDM simulation type, absorbing element and edge. *Web-CONEXS* then fixes several parameters automatically to ensure the feasibility of the simulation on the IRIS compute cluster: the cluster sizes surrounding the absorbing atom are fixed at 6 Å; moreover, the spectrum is computed in the energy range −10 eV ≤ *E* ≤ 50 eV surrounding the Fermi level, expanding the electronic transition operator up to the quadrupole approximation.

## 3. Case studies

### 3.1. Pre-edge features in spin-crossover complexes

**Figure 2:**
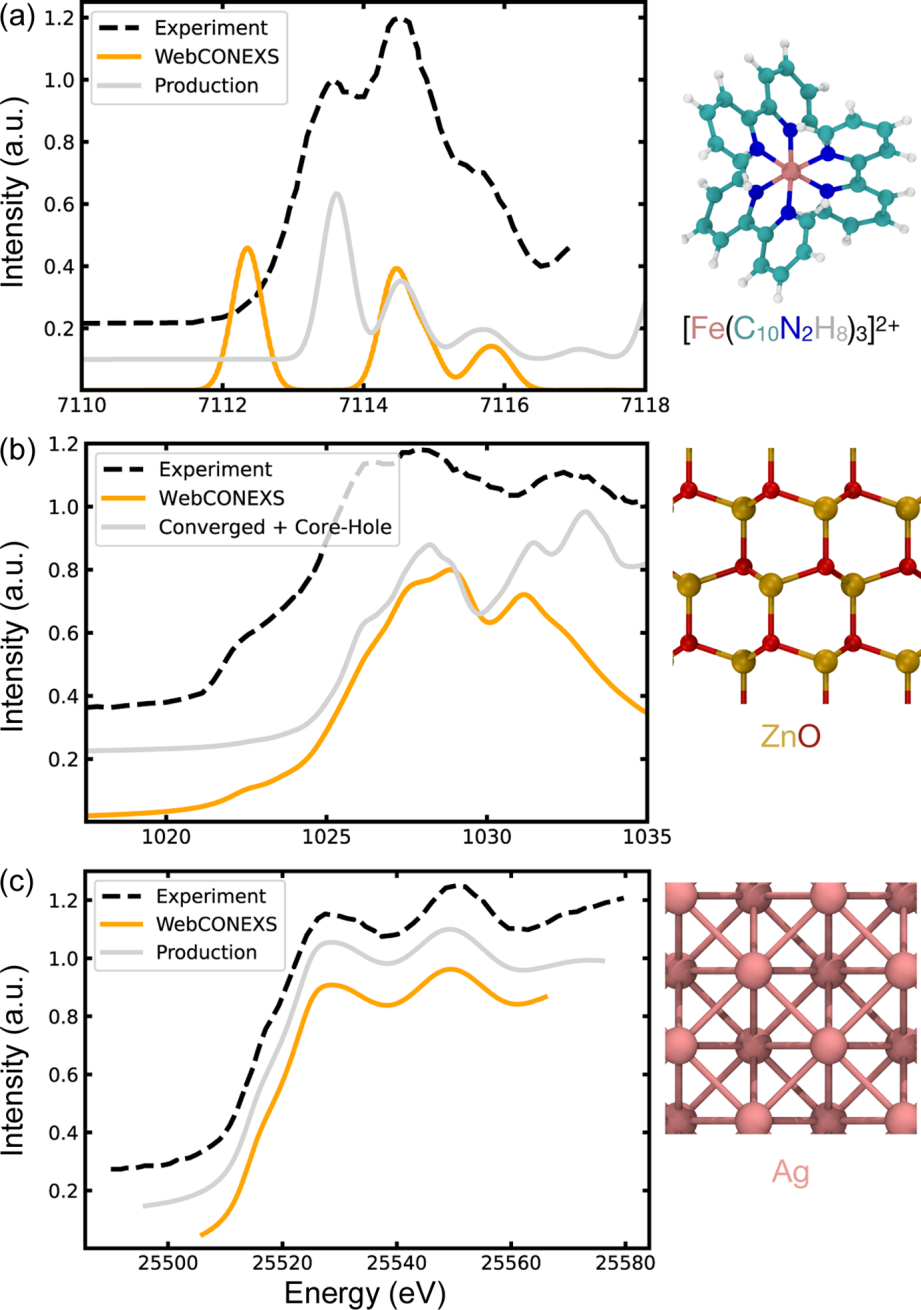
Results of the case studies conducted using the *Web-CONEXS* platform. (*a*) Plot comparing the theoretical and experimental Fe *K*-edge for the organometallic complex [Fe(bpy)_3_]^2+^ in the low-spin case. (*b*) Plot comparing the Zn *L*_3_-edge of ZnO measured experimentally computed with *Web-CONEXS* and with fully converged parameters. (*c*) Plot of the Ag *K*-edge XANES spectra of Ag nanoparticles measured experimentally and computed using the *FDMNES* code. In all cases, experimental spectra are plotted with a dashed black line, the *Web-CONEXS* result is plotted with solid orange lines and an ‘accurate’ calculation is plotted with a solid grey line.

The *ORCA* software package can be utilized for the investigation of molecular systems; we used the *Web-CONEXSORCA* interface to simulate the pre-edge features of the Fe *K*-edge in the organometallic spin crossover complex [Fe(bpy)_3_]^2+^. The geometry of the [Fe(bpy)_3_]^2+^ complex is depicted in Fig. 2 ▸(*a*), the Fe^2+^ ion is octahedrally coordinated, with the five 3*d* orbitals split by crystal field effects into the *e*_*g*_ and *t*_2*g*_ sets. Fe^2+^ has the valence electron configuration 3*s*^2^3*p*^6^3*d*^6^4*s*^0^; in the low-spin (LS) configuration, the *t*_2*g*_ set is filled while the *e*_*g*_ set is empty. This means that the pre-edge features in the Fe *K*-edge can arise from 1*s* → 3*d* transitions to the empty *e*_*g*_ set and metal-to-ligand charge transfer (MLCT) excitations where there is sufficient spatial overlap of the Fe and ligand π-orbitals.

Fig. 2 ▸(*a*) reports the high-energy-resolution fluorescence-detected (HERFD) pre-edge spectrum with a dashed black line (Capano *et al.*, 2013 ▸). Three features are shown: firstly, a peak at 7113.5 eV, which is attributed to a Fe 1*s* → 3*d* transition; and a second peak and shoulder at 7114.5 and 7115.5 eV, respectively, that arise from MLCT excitations (Capano *et al.*, 2013 ▸).

We initialized the *Web-CONEXS* calculation, first selecting to perform an XAS calculation with the B3LYP hybrid exchange-correlation functional and def-SVP basis set. The total charge for the calculation was set to 2*e*, while the multiplicity (2*S* + 1, with *S* equal to the total spin) in the LS configuration is 1. The geometry, previously determined by EXAFS, is imported directly from an .xyz file. We selected ABSQ to obtain the absorption spectrum, which means that the computed spectrum will include the electric dipole and quadrupole transitions, with an 0.5 eV Gaussian broadening in the relevant energy range to the Fe *K*-edge. In Fig. 2 ▸(*a*), the theoretical pre-edge, computed based on *Web-CONEXS* parameters, is plotted with a solid orange line. We have applied a rigid shift to the spectrum energy *a posteriori*, which accounts for a systematic underestimation of the core-electron binding energy. To help guide the eye we have chosen the magnitude of the shift to align the second peak of the theoretical and experimental spectra since the relative energy separation of the two MLCT excitations is well reproduced. *Web-CONEXS* reproduces the two peaks and shoulder features observed in the experimental measurement; in particular, the relative position of the MLCT peak and shoulder are well described. The peak corresponding to the 1*s* → 3*d* transition is red-shifted by approximately 1 eV compared with the experiment, due to neglect of electron correlation in Hartree–Fock theory which over-stabilizes the 3*d*(*e*_*g*_) orbitals (Reiher *et al.*, 2001 ▸) as discussed by Capano *et al.* (2013 ▸).

Systematic improvements, building on the input parameters generated by *Web-CONEXS*, lead to better agreement with the experiment. These improvements may be carried out (independently from the *Web-CONEXS* platform) by the user; this requires access to a computing cluster and resubmission of the (updated) input files using a job script. The grey solid line plotted in Fig. 2 ▸(*a*) is the result of a simulation with improved (but more computationally demanding) input parameters, which we define as production level. In this case, the energetic alignment of all three of the pre-edge features is found to agree with the experimental spectrum. For the computation of this spectrum, we first increased the size of the basis set, opting to use TZVP. Next, following Capano *et al.* (2013 ▸), we tuned the fraction of exact exchange contained in the B3LYP functional. A Hartree–Fock exchange of 12.5% was found to reproduce the peak-splitting observed in the experiment. Additionally, we computed the first 50 core excitations to ensure convergence in the edge region. Finally, we applied a Gaussian broadening with a full width at half-maximum equal to 0.5 eV. These changes are reflected in the orca.inp file provided in the supporting information.

### 3.2. *L*-edge spectra in crystalline powders

To demonstrate the capabilities of the *Web-CONEXSQE* interface, we computed the Zn *L*_3_-edge of ZnO. This well defined crystalline material adopts the hexagonal wurtzite structure with the space group *P*6_3_*mc*. Within the 3*d* lattice, Zn^2+^ ions are ionically bonded to O^2−^, which leads to them having a complete 3*d* shell and the valence electron configuration 3*s*^2^3*p*^6^3*d*^10^4*s*^0^. Transitions at the Zn *L*_3_ edge correspond to Zn 2*p* → Zn 4*s* and Zn 2*p* → Zn 4*d* excitations. Fig. 2 ▸(*b*) plots the Zn *L*_3_ edge for ZnO powder (Primo *et al.*, 2022 ▸) in dashed black lines. The main absorption edge is a broad feature with at least two peaks centred at 1026.5 eV and 1028 eV. A second broad absorption feature is observed at higher energy, with a maximum at 1032.5 eV. Finally, there is a shoulder in the rising edge located at 1022.5 eV. The low-energy shoulder is assigned to Zn 2*p* → Zn 4*s* transitions, while the higher energy peaks are considered to be due to Zn 2*p* → Zn 4*d* transitions (Primo *et al.*, 2022 ▸).

To set up the ZnO system within *Web-CONEXS*, we first imported the structure using the Materials Project identification number MP-2133 (Jain *et al.*, 2013 ▸; Ong *et al.*, 2015 ▸), opting for the *L*_3_ absorption edge and selecting Zn as the absorbing species. In Fig. 2 ▸(*b*), the *Web-CONEXS* computed spectrum is plotted with a solid orange line. The theoretical line shape broadly agrees with the experimental measurement, demonstrating how the software can be utilized for preliminary modelling of the XAS spectrum. The edge is reproduced with an approximate 1.5 eV difference between the two absorption peaks, close to the observed experimental value. The second broad signal is present but red-shifted to 1031 eV and is significantly narrower than in the experimental data.

In all instances, the input and output provided by *Web-CONEXS* for *QE* should be a starting point for further production-level calculations of the XANES spectrum. To highlight this, a fully converged simulation of the Zn *L*_3_-edge is shown in grey in Fig. 2 ▸(*b*). Careful consideration of the various input parameters leads to a theoretical spectral line shape that qualitatively agrees with the experimental measurement over the entire energy range. As with the *Web-CONEXS* result, the energy-splitting of the peaks in the absorption edge is well reproduced (1.5 eV). Moreover, a shoulder at 1028.3 eV emerges as an additional feature beyond the resolution of the reference experimental data. The energetic alignment of the second broad feature is also greatly improved, with the simulated spectrum suggesting the presence of two transitions at 1031 eV and 1032.5 eV. The Zn 2*p* → Zn 4*s* low-energy shoulder is not captured in these simulations, possibly due to the approximate treatment of many-body excitonic effects via a static core-hole potential within the underlying theoretical framework.

To obtain this fully converged simulation, starting from the *Web-CONEXS* input, we carried out convergence tests on the kinetic energy cutoff of the plane-wave basis set and the density of the **k**-point mesh used to sample the first Brillouin zone during the SCF and XANES calculation steps. We found that a 70 Ry cutoff on plane waves and a 6 × 6 × 6 **k**-point grid sufficient to converge simulations of the primitive cell. To approximate the many-body effects due to the presence of a core hole, we constructed a Zn pseudopotential with a hole in the *2p* level and added a total charge of +1.0 *e* to the simulation cell. Artificial interactions between periodic replicas of the core-hole were removed using a 3 × 3 × 3 supercell with a **k**-point grid scaled to 3 × 3 × 3 points. To construct the powder spectrum, we repeated the XANES calculation, averaging over the three polarization directions. A constant Gaussian broadening of 0.4 eV was applied to the spectral range. All files related to these simulations are provided in the supporting information.

### 3.3. *K*-edge spectra for Ag nanoparticles

As an example using the *FDMNES* interface, we have computed the Ag *K*-edge absorption spectrum for bulk Ag, which can exist within Ag nanoparticles (Godfrey *et al.*, 2020 ▸). The crystal structure of Ag is presented in Fig. 2 ▸(*c*); it adopts an f.c.c. structure with the space group *Fm*3*m*. Each Ag^0^ has the valence electron configuration 4*s*^2^4*p*^6^4*d*^10^5*s*^1^, leading to a *K*-edge spectrum characterized by 1*s* → 5*p* transitions. Fig. 2 ▸(*c*) reports the experimental Ag *K*-edge XANES spectrum for Ag foil with a black dashed line (Godfrey *et al.*, 2020 ▸). The spectrum has a shoulder in the rising edge at 25517 eV. The main peak is broad, with a maximum at 25527 eV and there is a second, broad and more intense peak at 25551 eV.

We set up the Ag crystal on the *Web-CONEXSFDMNES* interface using a .cif obtained from entry mp-124 of the Materials Project database (Jain *et al.*, 2013 ▸; Ong *et al.*, 2015 ▸). We then selected to compute the *K*-edge for Ag, opting to treat the material as a crystal. The *Web-CONEXS* result is plotted with a solid orange line in Fig. 2 ▸(*c*). The simulated spectrum is in good agreement with the experimental measurement. In particular, the energetic alignment (25516 eV, 25528 eV and 25549 eV), energy range, and intensity of the shoulder and two peaks all match their experimental counterparts.

Even though, in this case, the *Web-CONEXS* result satisfactorily reproduces the experimental spectrum, we performed a series of further simulations with the aim of demonstrating how the basic input parameters provided by the platform may be improved. The resulting simulation, which is plotted with a solid grey line in Fig. 2 ▸(*c*), similar to the *Web-CONEXS* result, reproduces all of the main features of the spectrum over a larger energy range. To obtain the final simulated spectrum, we included spin-orbit interactions and set the electronic occupancy of the spin-up and spin-down valence channels to 4*d*^10^5*s*^0.5^. Furthermore, we increased the radius of the cluster where the final state calculation is performed to 8 Å and increased the energy range for the calculation to −20 eV ≤ *E*_F_ ≤ 60 eV. All files related to these simulations are provided in the supporting information.

## 4. Conclusions

Computational core-hole spectroscopy can be instrumental in characterizing the electronic transitions observed in an experimental X-ray spectra. For non-specialist users, theoretical X-ray simulations based on electronic structure theory require a significant (and prohibitive) investment in time and resources. Here, we introduced *Web-CONEXS*, a web application hosted on *ISPyB* at DLS, which provides a platform for modelling the XAS spectra based on minimal user input.

*Web-CONEXS* interfaces the three different electronic structure theory codes, each with well defined uses. For molecular systems and cluster-type simulations, *Web-CONEXS* interfaces to the *ORCA* package; for periodic and crystalline systems, *Web-CONEXS* interfaces to the *QE* software suite; and for large computationally demanding systems, *Web-CONEXS* interfaces to *FDMNES*.

*Web-CONEXS* offsets complexities associated with creating code-specific input files by generating minimal viable input that serves as a starting point for the user based on their material of interest. In addition, *Web-CONEXS* handles the submission of batch jobs to the High-Throughput Compute facility IRIS, and the retrieval and post-processing of simulation output, further reducing the burden on the user.

To demonstrate the efficacy of the approach, we applied *Web-CONEXS* to three different user cases: (i) the pre-edge features of an organometallic spin-crossover complex in the hard X-ray regime, (ii) the *L*-edge powder spectrum of a crystalline transition metal oxide in the soft X-ray regime and (iii) the *FDMNES* example. In each example, we demonstrated how *Web-CONEXS* can be utilized as a relatively good starting point for modelling the spectrum and how to build on the generated input to obtain production-level simulations in agreement with the experimental measurements.

## Supplementary Material

Simulation parameters and software input files. DOI: 10.1107/S1600577524005630/ok5117sup1.pdf
