# Supplementary material for: Web-CONEXS: an inroad to theoretical X-ray absorption spectroscopy
Source: J Synchrotron Radiat. 2024 Aug 1;31(Pt 5):1276–84. doi: 10.1107/S1600577524005630 (PMC11371047; doi:10.1107/S1600577524005630)
Supplement: Supplementary file 1 [file s-31-01276-sup1.pdf]

JOURNAL OF
SYNCHROTRON
RADIATION

Volume 31 (2024)

Supporting information for article:

Web-CONEXS: an inroad to theoretical X-ray absorption spectroscopy

Joshua D. Elliott, Victor Rogalev, Nigel Wilson, Mihai Duta, Christopher J. Reynolds, Jacob Filik, Thomas J. Penfold and Sofia Diaz-Moreno

Supplementary Material for
Web-CONEXS: An inroad to theoretical x-ray absorption spectroscopy.

Joshua D. Elliott,[1,*] Victor Rogalev,[1] Nigel Wilson,[1] Mihai Duta,[1] Christopher J. Reynolds,[1] Jacob Filik,[1] Thomas J. Penfold,[2,*] and Sofia Diaz-Moreno[1,*]

[1]*Diamond Light Source, Harwell Science and Innovation Campus, Didcot, Oxfordshire OX11 0DE, United Kingdom*

[2]*Chemistry – School of Natural and Environmental Science, Newcastle University, Newcastle Upon-Tyne, NE1 7RU, United Kingdom*

I. WEB-CONEXS PARAMETERS.

This section of the electronic supplementary information details the input keywords and the available options for each of the calculators on the Web-CONEXS platform. It is important to stress that the input generated by Web-CONEXS (based on these selections) should only be used as a starting point for the simulation of the spectra, and should be followed by appropriate convergence testing.

Parameter	Web-CONEXS Allowed Value	Web-CONEXS Default
**Orca**		
Exchange-Correlation Functional	BLYP .OR. B3LYP	BLYP
Basis Set	def2-SVP .OR. def2-SV(P)	def2-SVP
Total Charge	Total nuclear charge - total electron charge	0
Spin Multiplicity	Value from $2S + 1$	1
Solvent	Name of solvent	None
Orbital Excitation Window	Core-orbital range, virtual orbital range	0, 0, -1, -1
**Quantum ESPRESSO**		
Basis Set Cutoff	50 Ry	50 Ry
Occupations	'fixed' (semiconductor/insulator) .OR. 'smearing' (metallic)	'smearing'
Smearing mode	'Fermi-Dirac' (metallic)	'Fermi-Dirac'
Smearing width	5×10^{-3} Ry (metallic)	5×10^{-3} Ry
Density mixing	0.2	0.2
**k**-point mesh	1 1 1 0 0 0	1 1 1 0 0 0
**FDMNES**		
Edge	Value from K to M5	K
Simulation Method	Finite Difference .OR. Green's Function	Finite Difference
System type	Crystal .OR. molecule	crystal
Cluster Radius	6 Å	6 Å

II. CASE STUDY: PRE-EDGE FEATURES IN SPIN-CROSSOVER COMPLEXES.

This section of the electronic supporting information reports the final input files required for the calculation of the pre-edge features in the Fe K-edge of the $[Fe(bipy)_3]^{2+}$ complex. The input is separated into two subsections, one listing the various input parameters and the second reporting the atomic coordinates.

A. ORCA input file

```
! B3LYP DKH2 TZVP
%maxcore 5024

%method
ScalHFX = 0.125
end

%pal nprocs 4
end

%tddft
orbWin[0] = 0,0,-1,-1
orbWin[1] = 0,0,-1,-1
```

```
doquad true
nroots 50
maxdim 10
end

* xyzfile 2 1 input.xyz
```

B. Input geometry

```
 61

 Fe  19.6126618831583      20.1420232390773      18.59820532054135
  N   21.1163195060722      20.8636013682709      19.64046230763085
  N   21.0772552645937      19.3437656752872      17.55736967793789
  N   19.4932029664519      18.4606655280346      19.60781979447754
  N   18.1792042849229      19.2938597223474      17.55250479001350
  N   18.2237309864545      21.0614041505474      19.64335072022072
  N   19.5807462803218      21.8269485490746      17.58889163296992
  C   21.0300215143113      21.6930020652400      20.70872055941307
  H   20.0190568539261      21.9514243350336      21.05170137056028
  C   22.1550611901447      22.2030180278056      21.36496376313318
  H   22.0219499304044      22.8700440005342      22.22875370639184
  C   20.3666955538740      22.1460993380988      16.53200102507076
  H   21.0584269696125      21.3688369201831      16.18051269670577
  C   23.4326074313935      21.8503364798751      20.90313757716371
  H   24.3381627486830      22.2353137185336      21.39455429180704
  C   23.5316379264323      20.9978602826743      19.79802396475687
  H   24.5180181822862      20.7086158847773      19.41066233990819
  C   22.3618202118629      20.5171001072126      19.18221835476067
  C   22.3385574053740      19.6180994646456      18.02102003521835
  C   23.4814761372512      19.0717906179191      17.40932339305235
  H   24.4812646529296      19.3035309326003      17.80094714563810
  C   23.3383701169276      18.2275411056859      16.30269771341712
  H   24.2223748558749      17.7915368859049      15.81467977192731
  C   22.0447265856860      17.9499098152611      15.83451860593833
  H   21.8771416001540      17.2930898127335      14.96890243402273
  C   20.9480666574666      18.5227797920319      16.48683622005500
  H   19.9254781371682      18.3243460309866      16.13837456191222
  C   20.2622500679550      18.1010738909879      20.66409649664022
  H   20.9949874067957      18.8405480540692      21.01410968783068
  C   20.1428957420002      16.8618247154758      21.30161725350640
  H   20.7938008907002      16.6279615750771      22.15624620272347
  C   19.1910168774252      15.9427265801217      20.83354692199059
  H   19.0720853802434      14.9589223963462      21.31073788512905
  C   18.3943736202700      16.3040375388279      19.74146221720483
  H   17.6435720600820      15.6041199060469      19.35019146776197
  C   18.5626829370387      17.5662797618456      19.14393985432866
  C   17.7868231577515      18.0574963764636      17.99797353727999
  C   16.7362000068397      17.3530144197545      17.38232214036672
  H   16.4385456726410      16.3654103675086      17.76033692721663
  C   16.0706119403066      17.9181670461274      16.28907111493588
  H   15.2464379210192      17.3804175459109      15.79786963985189
  C   16.4778409433045      19.1831782314497      15.83785819112909
  H   15.9891081583549      19.6708061988052      14.98219088400410
  C   17.5278995323070      19.8339367940204      16.49389043137954
  H   17.8677836970786      20.8225593834011      16.15658768225923
  C   17.5436352580433      20.5552673988423      20.70065824313680
```

```
H   17.8282193625715      19.5484660673964      21.03518663245538
C   16.5319096386158      21.2616313400055      21.35953123302201
H   16.0172498025605      20.7993863364056      22.21407809125435
C   16.1960385063927      22.5489966781017      20.91279803119179
H   15.4041545160527      23.1309419965394      21.40672890594569
C   16.8911797601489      23.0790168657076      19.82029184458917
H   16.6488755876984      24.0827877621720      19.44555526400854
C   17.9000173843895      22.3187626794566      19.20133891757429
C   18.7001222930589      22.7692936458746      18.05529357390173
C   18.5992692048586      24.0395962938166      17.45958415255657
H   17.8875368582713      24.7785564691876      17.85218229409277
C   19.4127424029495      24.3590381239931      16.36685920055596
H   19.3461915789086      25.3484476243555      15.89107425690736
C   20.3132000820050      23.3907660220419      15.89611959805679
H   20.9744849176203      23.5904990634844      15.04079445056675
```

III. CASE STUDY: *L*-EDGE SPECTRA IN CRYSTALLINE POWDERS.

This section of the electronic supporting information reports the final input files required for the calculation of the Zn *L*-edge of the hexagonal ZnO crystal. The input is separated into three subsections, one for each step required in the simulation of the xanes and one for the generation of the Zn pseudopotential . We first report the input for a single-point SCF calculation for a $3 \times 3 \times 3$ ZnO supercell with one Zn atom modified to contain a core-hole; second we report the input for the calculation of the (polarisation dependent) XANES spectrum; and finally, the input needed to generate a Zn pseudopotential with a hole in the 2 p level, with one projector in each angular momentum channel.

A. Input file for pw.x

```
&CONTROL
 calculation = 'scf'
 etot_conv_thr = 1.0000000000d-05
 outdir = './TMP'
 prefix = ''
 pseudo_dir = './'
 restart_mode = 'from_scratch'
 title = ''
 tprnfor = .FALSE.
 verbosity = 'low'
 forc_conv_thr = 1.0000000000d-04
/

&SYSTEM
 ibrav = 0
 occupations = 'smearing'
 smearing = 'fermi-dirac'
 degauss = 5.0000000000d-03
 nat = 108
 ntyp = 3
 ecutwfc = 70
 tot_charge = 1.
/

&ELECTRONS
 diagonalization = 'david'
 electron_maxstep = 50
 mixing_beta = 0.2
/
```

```
ATOMIC_SPECIES
Zn* 65.38 Znstar2p.pbe-dn-rrkjus_gipaw.UPF
Zn 65.38 Zn.pbe-dn-rrkjus_gipaw.UPF
O 15.999 O.pbe-n-rrkjus_gipaw.UPF


ATOMIC_POSITIONS {angstrom}
Zn*      1.61867539     -0.93454267      2.61412035
Zn       1.61867539      0.93454267      0.00308989
O        1.61867539     -0.93454267      4.59394256
O        1.61867539      0.93454267      1.98291210
Zn       1.61867539     -0.93454267      7.83618127
Zn       1.61867539      0.93454267      5.22515081
O        1.61867539     -0.93454267      9.81600348
O        1.61867539      0.93454267      7.20497302
Zn       1.61867539     -0.93454267     13.05824219
Zn       1.61867539      0.93454267     10.44721173
O        1.61867539     -0.93454267     15.03806440
O        1.61867539      0.93454267     12.42703394
Zn       3.23735078      1.86908535      2.61412035
Zn       3.23735078      3.73817069      0.00308989
O        3.23735078      1.86908535      4.59394256
O        3.23735078      3.73817069      1.98291210
Zn       3.23735078      1.86908535      7.83618127
Zn       3.23735078      3.73817069      5.22515081
O        3.23735078      1.86908535      9.81600348
O        3.23735078      3.73817069      7.20497302
Zn       3.23735078      1.86908535     13.05824219
Zn       3.23735078      3.73817069     10.44721173
O        3.23735078      1.86908535     15.03806440
O        3.23735078      3.73817069     12.42703394
Zn       4.85602618      4.67271338      2.61412035
Zn       4.85602618      6.54179872      0.00308989
O        4.85602618      4.67271338      4.59394256
O        4.85602618      6.54179872      1.98291210
Zn       4.85602618      4.67271338      7.83618127
Zn       4.85602618      6.54179872      5.22515081
O        4.85602618      4.67271338      9.81600348
O        4.85602618      6.54179872      7.20497302
Zn       4.85602618      4.67271338     13.05824219
Zn       4.85602618      6.54179872     10.44721173
O        4.85602618      4.67271338     15.03806440
O        4.85602618      6.54179872     12.42703394
Zn       3.23735078     -3.73817069      2.61412035
Zn       3.23735078     -1.86908535      0.00308989
O        3.23735078     -3.73817069      4.59394256
O        3.23735078     -1.86908535      1.98291210
Zn       3.23735078     -3.73817069      7.83618127
Zn       3.23735078     -1.86908535      5.22515081
O        3.23735078     -3.73817069      9.81600348
O        3.23735078     -1.86908535      7.20497302
Zn       3.23735078     -3.73817069     13.05824219
Zn       3.23735078     -1.86908535     10.44721173
O        3.23735078     -3.73817069     15.03806440
O        3.23735078     -1.86908535     12.42703394
Zn       4.85602618     -0.93454267      2.61412035
Zn       4.85602618      0.93454267      0.00308989
```

O	4.85602618	-0.93454267	4.59394256
O	4.85602618	0.93454267	1.98291210
Zn	4.85602618	-0.93454267	7.83618127
Zn	4.85602618	0.93454267	5.22515081
O	4.85602618	-0.93454267	9.81600348
O	4.85602618	0.93454267	7.20497302
Zn	4.85602618	-0.93454267	13.05824219
Zn	4.85602618	0.93454267	10.44721173
O	4.85602618	-0.93454267	15.03806440
O	4.85602618	0.93454267	12.42703394
Zn	6.47470157	1.86908535	2.61412035
Zn	6.47470157	3.73817069	0.00308989
O	6.47470157	1.86908535	4.59394256
O	6.47470157	3.73817069	1.98291210
Zn	6.47470157	1.86908535	7.83618127
Zn	6.47470157	3.73817069	5.22515081
O	6.47470157	1.86908535	9.81600348
O	6.47470157	3.73817069	7.20497302
Zn	6.47470157	1.86908535	13.05824219
Zn	6.47470157	3.73817069	10.44721173
O	6.47470157	1.86908535	15.03806440
O	6.47470157	3.73817069	12.42703394
Zn	4.85602618	-6.54179872	2.61412035
Zn	4.85602618	-4.67271338	0.00308989
O	4.85602618	-6.54179872	4.59394256
O	4.85602618	-4.67271338	1.98291210
Zn	4.85602618	-6.54179872	7.83618127
Zn	4.85602618	-4.67271338	5.22515081
O	4.85602618	-6.54179872	9.81600348
O	4.85602618	-4.67271338	7.20497302
Zn	4.85602618	-6.54179872	13.05824219
Zn	4.85602618	-4.67271338	10.44721173
O	4.85602618	-6.54179872	15.03806440
O	4.85602618	-4.67271338	12.42703394
Zn	6.47470157	-3.73817069	2.61412035
Zn	6.47470157	-1.86908535	0.00308989
O	6.47470157	-3.73817069	4.59394256
O	6.47470157	-1.86908535	1.98291210
Zn	6.47470157	-3.73817069	7.83618127
Zn	6.47470157	-1.86908535	5.22515081
O	6.47470157	-3.73817069	9.81600348
O	6.47470157	-1.86908535	7.20497302
Zn	6.47470157	-3.73817069	13.05824219
Zn	6.47470157	-1.86908535	10.44721173
O	6.47470157	-3.73817069	15.03806440
O	6.47470157	-1.86908535	12.42703394
Zn	8.09337697	-0.93454267	2.61412035
Zn	8.09337697	0.93454267	0.00308989
O	8.09337697	-0.93454267	4.59394256
O	8.09337697	0.93454267	1.98291210
Zn	8.09337697	-0.93454267	7.83618127
Zn	8.09337697	0.93454267	5.22515081
O	8.09337697	-0.93454267	9.81600348
O	8.09337697	0.93454267	7.20497302
Zn	8.09337697	-0.93454267	13.05824219
Zn	8.09337697	0.93454267	10.44721173
O	8.09337697	-0.93454267	15.03806440
O	8.09337697	0.93454267	12.42703394

```
K_POINTS automatic
3 3 3 0 0 0


CELL_PARAMETERS {angstrom}
4.8560261825240225 -8.410884071016344 0.
4.8560261825240225  8.410884071016344 0.
 0 0 15.666182762280133
```

B. Input file for xspectra.x

```
&input_xspectra
calculation='xanes_dipole'
prefix=''
outdir = './TMP'
edge='L3'
xiabs=1
xepsilon(1)=1.0
xepsilon(2)=0.0
xepsilon(3)=0.0
!xepsilon(1)=0.0
!xepsilon(2)=1.0
!xepsilon(3)=0.0
!xepsilon(1)=0.0
!xepsilon(2)=0.0
!xepsilon(3)=1.0
xniter=1000
xcheck_conv=100
xerror=0.001
x_save_file='xanes_100.sav'
!x_save_file='xanes_010.sav'
!x_save_file='xanes_001.sav'
/
&plot
xnepoint=1000
xgamma=0.4
xemin=-10.0
xemax=30.0
terminator=.true.
cut_occ_states=.true.
xanes_file='l3_xanes_100.dat'
!xanes_file='l3_xanes_010.dat'
!xanes_file='l3_xanes_001.dat'
/
&pseudos
filecore='core.wfc'
r_paw(1)=3.2
/
&cut_occ
cut_desmooth=0.1
cut_stepl=0.01
/
3 3 3 1 1 1
```

C. Input file for ld1.x to generate Zn pseudopotential with 2 p core hole

```
&input
title='Zn',
zed=30.,
rel=1,
config='1s2 2s2 2p5 3s2 3p6 4s2 4p0.3 3d9.7',
iswitch=3,
dft='PBE'
/
&inputp
lgipaw_reconstruction = .true.
lpaw=.false.,
pseudotype=3,
file_pseudopw='Znstar2p.pbe-dn-rrkjus_gipaw.UPF',
lloc=-1,
rcloc=1.9,
which_augfun='PSQ',
rmatch_augfun_nc=.true.,
nlcc=.true.,
new_core_ps=.true.,
rcore=1.0,
tm=.true.
/
6
4S  1  0  2.00  0.00  1.20  2.20  0.0
4S  1  0  0.00  6.10  1.20  2.20  0.0
4P  2  1  0.30  0.00  1.20  2.20  0.0
4P  2  1  0.00  6.30  1.20  2.20  0.0
3D  3  2  9.70  0.00  1.20  1.70  0.0
3D  3  2  0.00 -0.80  1.20  1.70  0.0
```

IV. CASE STUDY: K-EDGE SPECTRA FOR AG-NANOPARTICLES.

In this section of the electronic supporting information we provide the FDMNES input file used to compute the Ag K-edge spectrum in the bulk Ag system.

```
Filout
result

Range
-20. 0.25 60       !E_min, step, E_intermediate, step ...

Edge
K

Z_absorber
47

SCF            !Performs self-consistent calculation
Energpho       !Output energy relative to the photon energy of absorbing atom

Quadrupole

Radius         ! Radius of the cluster where final state calculation is performed
8
```

```
Spinorbit

Atom
47 2 4 2 5. 5. 5 0 0.5 0.5

Crystal
  4.1043564 4.1043564 4.1043564 90. 90. 90.
1 0.0 0.0 0.0
1 0.5 0.5 0.0
1 0.5 0.0 0.5
1 0.0 0.5 0.5

Convolution

End
```
